# Deep Brain Stimulation Complications in Patients With Parkinson’s Disease and Surgical Modifications: A Single-Center Retrospective Analysis

**DOI:** 10.3389/fnhum.2021.684895

**Published:** 2021-06-11

**Authors:** Shuo Xu, Wenfei Wang, Si Chen, Qianqian Wu, Chao Li, Xiangyu Ma, Teng Chen, Weiguo Li, Shujun Xu

**Affiliations:** ^1^Department of Neurosurgery, Qilu Hospital of Shandong University and Institute of Brain and Brain-Inspired Science, Shandong University, Jinan, China; ^2^Key Laboratory of Brain Function Remodeling, Qilu Hospital of Shandong University, Jinan, China; ^3^Humanistic Medicine Research Center, Qilu Hospital of Shandong University, Jinan, China

**Keywords:** deep brain stimulation, Parkinson’s disease, complications, operation, hardware, surgical modifications

## Abstract

**Background:**

As a complication-prone operation, deep brain stimulation (DBS) has become the first-line surgical approach for patients with advanced Parkinson’s disease (PD). This study aimed to evaluate the incidence and risk factors of DBS-associated complications.

**Methods:**

We have reviewed a consecutive series of patients with PD undergoing DBS procedures to describe the type, severity, management, and outcome of postoperative complications from January 2011 to December 2018. Both univariate and multivariate analyses were performed to identify statistically significant risk factors. We also described our surgical strategies to minimize the adverse events.

**Results:**

A total of 225 patients underwent 229 DBS implantation procedures (440 electrodes), of whom 20 patients experienced 23 DBS-associated complications, including ten operation-related complications and 13 hardware-related ones. Univariate analysis elucidated that comorbid medical conditions (*P* = 0.024), hypertension (*P* = 0.003), early-stage operation (*P* < 0.001), and unilateral electrode implantation (*P* = 0.029) as risk factors for overall complications, or more specifically, operation-related complications demonstrated in the stratified analysis. In contrast, no risk factor for hardware-related complications was identified. Statistical significances of hypertension (OR = 3.33, 95% CI: 1.14–9.71, *P* = 0.027) and early-stage (OR = 11.04, 95% CI: 2.42–50.45, *P* = 0.002) were further validated via multivariate analysis. As the annual number of DBS procedures increased, the incidence of complications gradually decreased (*R* = −0.699, *P* < 0.01). Additionally, there was a strong correlation between surgical complications and unplanned readmission (*R* = 0.730, *P* < 0.01).

**Conclusion:**

The importance of cumulative experience and relevant technique modifications should be addressed to prevent DBS-associated complications and unplanned readmission.

## Introduction

Deep brain stimulation (DBS) is the therapeutic approach of intracranial electrical stimulation, which uses a four-contact stimulating electrode stereotactically implanted in the target and connected via a subcutaneous wire to an implantable pulse generator (IPG) that is placed on the chest wall underneath the collarbone. Most targets of DBS are deep brain structures, including deep nucleus and white matter tracts (Herrington et al., 2016). By preventing the transmission of pathologic bursting and improving the processing of sensorimotor information, DBS results in a considerable reduction in various symptoms of movement disorders, including tremor, bradykinesia, and stiffness (Miocinovic et al., 2013).

As a minimally invasive, effective, reversible, and controllable approach, DBS has gradually replaced conventional destructive surgery and has become the most common surgical option for advanced Parkinson’s disease (PD)(Okun, 2012; Ramirez-Zamora and Ostrem, 2018). In most cases, the overall benefits of DBS surgery greatly outweigh its risks. Despite the good tolerability and safety of this therapeutic approach, a broad range of DBS-related complications have arisen, which marks it a complication-prone operation (Boviatsis et al., 2010; Rughani et al., 2013; Kantzanou et al., 2021).

In this retrospective analysis, we reviewed the demographic and clinical features of 225 patients who underwent DBS surgery in our center over an 8-year period (2011–2018) to analyze the incidence and risk factors for DBS-related complications and summarize certain surgical strategies to minimize such adverse events.

## Methods

### Patients and Clinical Data

The patients with PD who had undergone the DBS procedure in our center from January 2011 to December 2018 were retrospectively analyzed. Of note, a DBS procedure was defined as any stereotactic surgery that involved implantation of the new intracranial electrode(s) as well as IPG. A total of 229 DBS procedures of 225 patients were enrolled, and data was cross-checked with the manufacturers’ records (PINS Medical, Beijing, China and Medtronic, MN, United States). All DBS operations were performed by two primary surgeons (SjX and WL). Follow-up was done at the outpatient service and telephone interview by December 31, 2020. Readmission referred to the unplanned admission to our center for any clinical situation related to DBS procedure after the primary discharge. Appointed readmissions for IPG exchange and contralateral electrode implantation were excluded. Ethical approval was obtained from the Medical Ethical Committee, Qilu Hospital of Shandong University (KYLL-2019-1-066).

### DBS Procedure

Surgery was performed following our standard procedure protocol (Ma et al., 2017). In short, we performed a positioning MRI scan with Gadolinium-based contrast prior to surgery. These images are then fused with the Leksell Model G stereotactic frame (Elekta AB, Stockholm, Sweden) based on CT images acquired on the day of surgery. Microelectrode recording (MER) was conducted for intraoperative electrophysiological localization of subthalamic nucleus (STN) or globus pallidus internus (GPi) without sedative agents. The electrode placement was further confirmed via macro-stimulation. The IPG was then implanted into the subclavian pouch under general anesthesia. The electrodes were connected to the IPG through corresponding extension wires. The position of the electrodes was examined again in a postoperative CT review within 24 h.

### Modified Surgical Technique

**FIGURE 1:**
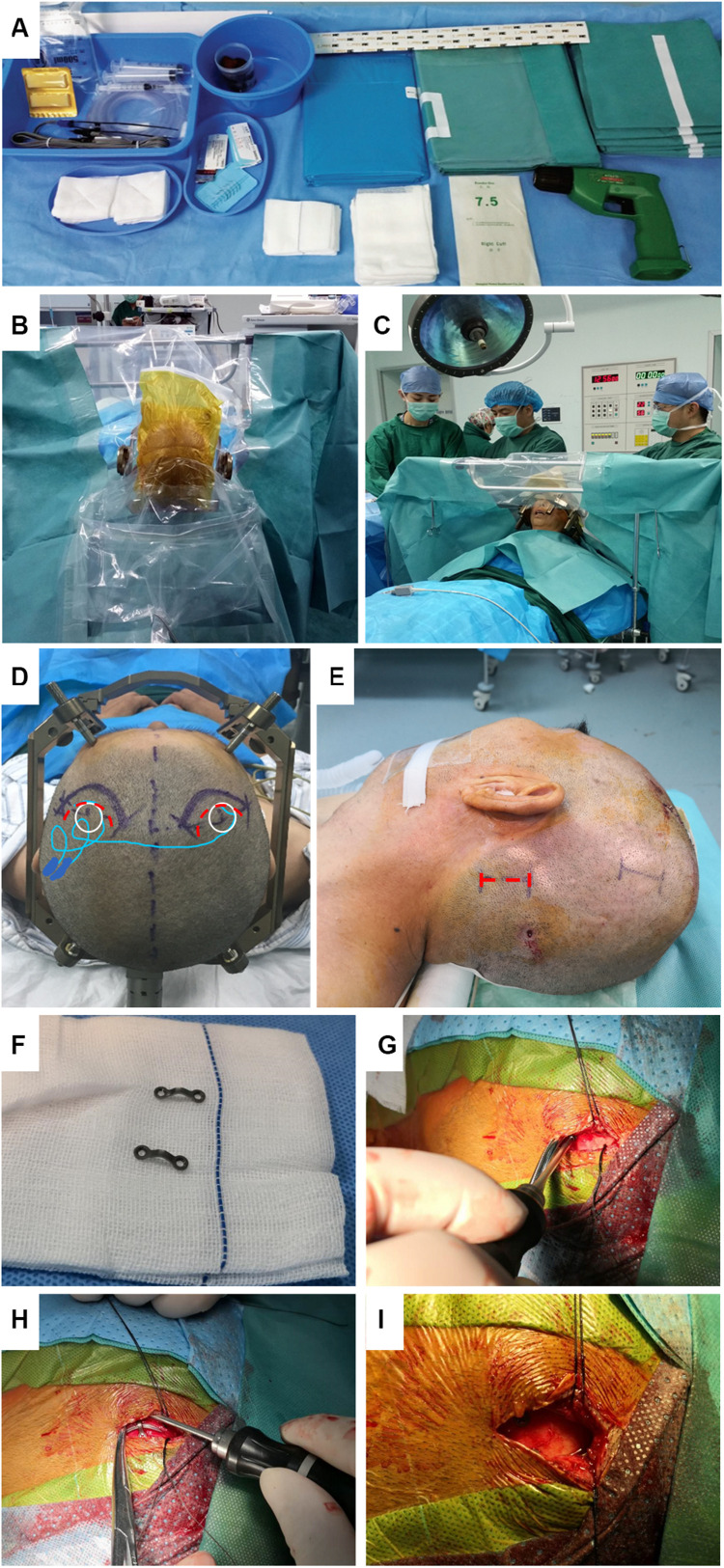
DBS Surgical modificationsin our center. **(A)** Demonstration of the non-woven drape exclusive for DBS surgery. **(B,C)** Transparent polyethylene film was easy to use and facilitated patient-surgeon communication during surgery. **(D)** Compared to the original incision on the coronal suture (red dotted curve), the distance between our modified forward-moving incision (purple solid curves) and burr hole and incision was increased to avoid placing hardware directly under the suture line. **(E)** The incision for the extension wire was moved upwards from occipital/mastoid region (red dotted line) to parietal eminence region (purple solid line) with thick skin and no lymph nodes. **(F)** Titanium microplate was bent to constrain the connector. **(G,H)** The skin was retracted aside so that the microplate was easily fixed to the skull. **(I)** Direct extension wire implantation under the suture line should be meticulously avoided.

Based on the literature review and our practical experience, we have gradually adopted several surgical modifications and summarize them as follows: (1) Special drapes for DBS surgery. The one-piece design with an integrated observation window was easy to use and facilitated patient-surgeon communication. The impermeable non-woven textile met the recommendation of WHO Guidelines for the prevention of surgical site infection (World Health Organization (WHO), 2018) (Figures 1A–C). (2) Frontal incisions. Bilateral C-shaped frontal incisions were performed about 1 inch ahead of the burr holes on the coronal suture so that the incisions would not overlie the electrode anchoring devices. This modification effectively prevented cable damaging and lowered the local scalp tension, which might cause delayed healing and infection (Figure 1D). (3) Extension wire fixation. The incision for the extension wire connection was moved upwards from the posterior to the pinna to the parietal eminence to shorten the distance to the frontal incisions. Scalp skin near the parietal eminence was thick and lacking lymph nodes, which reduced skin erosion and infection incidence. Also, the connectors were constrained with a remodeled 2-hole titanium microplates to prevent the migration (Figures 1E–I). (4) IPG fixation. The prepectoral subfascial pocket was created for the IPG implantation to minimize the local exudate and skin erosion observed in the subcutaneous pocket. The IPG was then fixed to the pectoralis muscle or the clavicle to avoid device migration.

### Statistical Analysis

Statistical analysis was performed using IBM SPSS Statistics for Windows (Version 22.0. IBM Corp., Armonk, NY, United States). The significance level was set at *P* < 0.05. Intergroup analysis was performed by either Student’s t-test or a combination of the chi-square test and Fisher exact test. Intergroup correlation was analyzed by the Spearman test. The risk factors were analyzed by multivariate logistic regression analysis using selected factors with a statistically significant difference in the univariate analysis. Graphs were drawn using Prism6 (GraphPad, United States). All the data was carefully reviewed by an experienced statistician (WW).

## Results

### Demographic and Clinical Features

From January 2011 to December 2018, 229 DBS procedures were performed for 225 patients with PD in our center. A total of 440 electrodes and 238 IPGs were implanted. At the same observation window, nine patients with dystonia, essential tremor, Meige’s syndrome, Tourette’s syndrome, and chorea underwent DBS surgery, and 39 patients with PD underwent 42 procedures of IPG replacement surgery in our center. Considering the limited sample size, these cases were then excluded from the following study aimed to analyze the DBS-associated complications for patients with PD.

As shown in Table 1, male patients accounted for 59.6% (134/225). The average age at surgery was 61.6 ± 7.9 years (range from 30 to 82 years), with the disease duration of 9.9 ± 4.8 years. Most of the patients (222/225) who underwent DBS procedures were Han Chinese. Among the 73 patients (32.4%) who presented with at least one comorbid condition, 48 (21.3%) had high blood pressure. 38 patients (16.9%) were with smoking history. Nearly two-thirds of PD patients (63.6%) received the surgical procedures in 2017-2018. Bilateral STNs were the dominant implantation targets (96.4%). About three-quarters majority (75.6%) of the newly implanted IPGs were manufactured by PINS Medical Inc., and the rest by Medtronic. Reasons for the second-time implantation procedures included the aborted procedures (2 cases), contralateral electrode implantation (1 case), and electrode misplace (1 case). At the end of December 2020, all patients were followed up for at least 24 months, with an average follow-up duration of 45.2 ± 17.7 months.

**TABLE 1:** Demographics of Patients with Postoperative Complications.

	**Total (*n* = 225)**	**Patients with complications (*n* = 20)**	**Patients without complications (*n* = 205)**	***p*-value**	**Statistical methodology**
**Age at surgery**	61.6 ± 7.9	62.3 ± 9.1	61.6 ± 7.8	0.70	Student’s t
**Disease history**	9.9 ± 4.8	9.9 ± 4.1	10.0 ± 4.9	0.93	Student’s t
**Gender**				0.66	Chi-square
Male	134(59.6%)	11(55%)	123(60%)		
Female	91(40.4%)	9(45%)	82(40%)		
**Ethnicity**				1	Fisher’s Exact Test
Han Chinene	222	20	202		
Non-Han Chinese	3	0	3		
**Comorbid conditions**					
** ≥ 1 comorbidity**	73(32.4%)	11(55%)	62(30.2%)	0.024	Chi-square
**≥ 2 comorbidities**	21	2	19	> 0.99	Chi-square (Continuity Correction)
Hypertension	48	10	38	0.003	Chi-square (Continuity Correction)
Diabetes	24	1	23	0.63	Chi-square (Continuity Correction)
Heart Disease	15	1	14	> 0.99	Chi-square (Continuity Correction)
Stroke	8	0	8	1	Fisher’s Exact Test
COPD/Asthma	3	1	2	0.25	Fisher’s Exact Test
**Smoking status**				0.94	Chi-square
**Smoker**	38	4	34		(Continuity Correction)
**Non-smoker**	187	16	171		
**PD duration**				0.66	Chi-square
** < 10yrs**	123	10	113		
** ≥ 10yrs**	102	10	92		
**Stage**				< 0.001	Chi-square
**Early (2011-2016)**	82	15	67		
**Late (2017-2018)**	143	5	138		
**Manufacturer**				> 0.99	Chi-square
**Medtronic**	55	5	50		(Continuity Correction)
Soletra	3	1	2		
Kinetra	1	1	0		
Activa PC	3	0	3		
Activa SC	1	0	1		
Activa RC	47	3	44		
**PINS Medical**	170	15*	155		
G101	13	3	10		
G101A	5	1	4		
G102	14	2	12		
G102R	132	8	124		
G102RZ	4	0	4		
G106R	2	1	1		
**Targets**				> 0.99	Fisher’s Exact Test
**STN**	217	20#	197		
**GPi**	8	0	8		
**Laterality of implant**				0.029	Chi-square
**Unilateral**	14	4&	10		(Continuity Correction)
Right	5	2&	3		
Left	9	2	7		
**Bilateral**	211	16	195		
**Readmission**				< 0.001	Chi-square
**Yes**	15	13	2		(Continuity Correction)
**No**	210	7	203		

*two patients whose IPG implantation procedures were terminated due to respiratory distress during the first operation; #one patients whose electrode implantation procedure was terminated due to respiratory distress during the first operation; &one patients whose contralateral electrode implantation was terminated due to respiratory distress after the implantation of the right electrode. Abbreviations: COPD, chronic obstructive pulmonary disease; GPi, globus pallidus internus; PD, Parkinson’s disease; STN, subthalamic nucleus.

### Complication Events and Categories

Generally, operation-related complications are defined as those that could potentially be prevented by a change in DBS surgical technique and hardware-related complications as they are more difficult to relate to surgical technique (Ramayya et al., 2017). In our series, 23 complications were observed in 20 patients, including 10 operation-related complications in nine patients and 13 hardware-related complications in 13 patients (shown in Table 2).

**TABLE 2:** Causes and interventions of complications.

**Patient**	**Gender**	**Age**	**PD history**	**Comorbidity**	**Operation date**	**Category**	**Complication details**	**Interventions**
1	F	52	17	Hypertension	2011/4/8	Operation	Electrode misplace	Re-operation (L-STN→bi-GPi) on 2017/4/28
2	M	49	7	Hypertension	2012/2/22	Operation	Severe peri-electrode edema	Recovery after symptomatic treatment
7	M	73	15		2013/9/13	Operation; Operation	Intracranial hematoma; epileptic seizure	Recovery after symptomatic treatment
12	M	57	7	Hypertension	2014/4/17	Operation	Peri-electrode edema with transient loss of consciousness	Recovery after symptomatic treatment
17	M	56	8	Hypertension	2014/8/25	Operation; Hardware	Electrode misplace; Extension wire high resistance	Removal of bilateral IPGs and extension wires on 2018/3/31
21	M	64	5	Hypertension	2014/12/4	Operation	Respiratory distress	Re-operation on 2016/1/24
29	F	62	14	Hypertension	2015/5/30	Hardware	Neck stricture formation	Under observation
36	M	65	10		2015/10/29	Hardware	Extension wire fracture	Replacement of IPG and bilateral extension wires on 2019/5/20
38	M	53	7	Hypertension	2015/11/19	Hardware	Extension wire fracture	Replacement of bilateral extension wires on 2017/12/1
44	M	64	10		2016/1/13	Hardware	Extension wire fracture after injury	Replacement of extension wire on 2018/4/3
59	F	68	10	Asthma	2016/8/15	Operation; Hardware	Respiratory distress; Chest subcutaneous exudate	Re-implantation of contralateral STN electrode and IPG on 2016/9/12 Recovery local compression dressing and vancomycin intravenous administration
61	M	70	7		2016/8/20	Operation	Hydrocephalus	ventriculoperitoneal shunt on 2017/10/13
66	F	55	10	Hypertension	2016/10/12	Hardware	Neck stricture formation	Relief of stricture on 2017/5/18
67	F	68	11		2016/10/20	Hardware	Electrode migration	Electrode adjustment on 2017/1/5
81	F	60	7		2016/12/29	Hardware	Electrode migration	Electrode adjustment on 2018/5/7
132	F	62	8		2017/11/16	Hardware	Chest subcutaneous exudate	Recovery without infection
160	M	43	4		2018/4/21	Hardware	Extension wire fracture after head injury	Replacement of right extension wire on 2019/6/12
171	F	72	6	Hypertension, Diabetes	2018/6/4	Hardware	IPG migration	Under observation
207	F	73	17		2018/10/12	Hardware	IPG migration	Under observation
217	M	79	17	Hypertension, Heart Disease	2018/12/2	Operation	Acute heart failure and pneumonia	Recovery after symptomatic treatment

*Abbreviations: GPi, globus pallidus internus; IPG, implantable pulse generator; PD, Parkinson’s disease; STN, subthalamic nucleus.*

**FIGURE 2:**
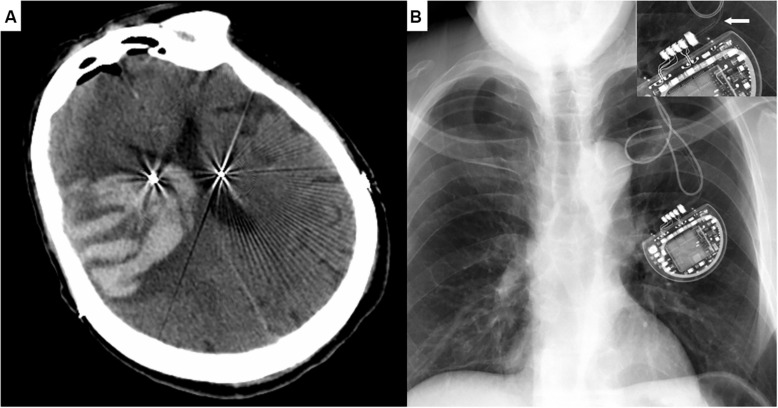
Representative Cases. **(A)** Cranial CT image of Patient #7 demonstrating the massive intracranial hematoma three days after the surgery, with symptoms of a generalized seizure. **(B)** Chest X-ray image of Patient #36 showing the fracture of extension wire near the IPG. Enlarged damaged wire in the right upper corner.

The observed operation-related complication included epileptic seizure combined with intracranial hematoma (Patient #7, Figure 2A), intraoperative respiratory distress (Patient #21, 59), severe peri-electrode edema (Patient #2, 12), electrode misplace (Patient #1, 17), acute heart failure (Patient #217) and hydrocephalus (Patient #61).

Wire fracture/high resistance was the most common hardware-related adverse event (Patient #17, 36, 38, 44, 160, as shown in Figure 2B). The others included electrode migration (Patient #67, 81), subcutaneous exudate/infection (Patient #59, 132), IPG migration (Patient #171, 207) and neck stricture formation (Patient #29, 66). Of note, two patients with subcutaneous exudate were categized into the minor infection, whom both recovered after local pressure and antibiotics administration. No etiological agent was diagnosed from the exudate laboratory examination.

As shown in Table 2, the outcomes were favorable under the appropriate interventions. No mortality or permanent morbidity was observed.

### Univariate Analysis

The univariate analysis revealed that patients with *hypertension* encountered complications more frequently (*P* = 0.003, Table 1). Given the high association with *hypertension* (*R* = 0.751, *P* < 0.01), *comorbid medical conditions* were also demonstrated as a risk factor (*P* = 0.024). Also, the complication rate of operation performed in 2011-2016 (hereinafter referred to as *early-stage*) was significantly higher than that in 2017-2018 (hereinafter referred to as *late-stage*, *P* < 0.001). Unexpectedly, the complication rate was higher in patients who received *unilateral electrode implantation* (*P* = 0.029), probably because of a weak but statistically significant association between *unilateral electrode implantation* and *early-stage* operation (*R* = 0.248, *P* < 0.01). No other variables were identified as risk factors. Of note, neither the primary diagnosis (PD or non-PD) nor surgical procedure (DBS or IPG replacement) appeared to affect the complication rates (*P* > 0.99 and P = 0.304, respectively).

Furthermore, a stratified analysis was conducted to elucidate the specific risk factors for operation- versus hardware-related complications. Notably, all these variables, including *comorbid conditions*, *hypertension* (*P* = 0.003), *early stage* (*P* = 0.003) and *unilateral implantation* (*P* = 0.006) were demonstrated as risk factors for operation-related complications. On the contrary, no potential predictors for hardware-related complications were identified (Table 3).

**TABLE 3:** Univariate Analysis of risk factors in complication subcategories with Chi-square Test with Continuity Correction.

	**Total (*n* = 225)**	**Patients with operation-related complications (*n* = 9)**	**Patients without operation-related complications (*n* = 216)**	***P*-value**
Hypertension	48	6	42	0.003
Early stage	82	8	74	0.003
Unilateral implant	14	3	11	0.006

	**Total (*n* = 225)**	**Patients with hardware-related complications (*n* = 13)**	**Patients without hardware-related complications (*n* = 212)**	***P*-value**

Hypertension	48	5	43	0.23
Early stage	82	6	76	0.65
Unilateral implant	14	2	12	0.41
Manufacturer				0.83
Medtronic	55	3	52	
PINS Medical	170	10	170	

### Multivariate Analysis

The variable *comorbid conditions* was excluded from the multivariate analysis because of its strong association with *hypertension*. The other three variables were then entered into the multivariate logistic regression analysis, which demonstrated *hypertension* (OR = 3.33, 95% CI: 1.14-9.71, *P* = 0.027) and *early stage* (OR = 11.04, 95% CI: 2.42-50.45, *P* = 0.002) as independent risk factors for postoperative complications. Although *unilateral implantation* was established as a univariate indicator of risk, its statistical significance was not demonstrated in the multivariate analysis (*P* = 0.074).

### Secular Trend

**FIGURE 3:**
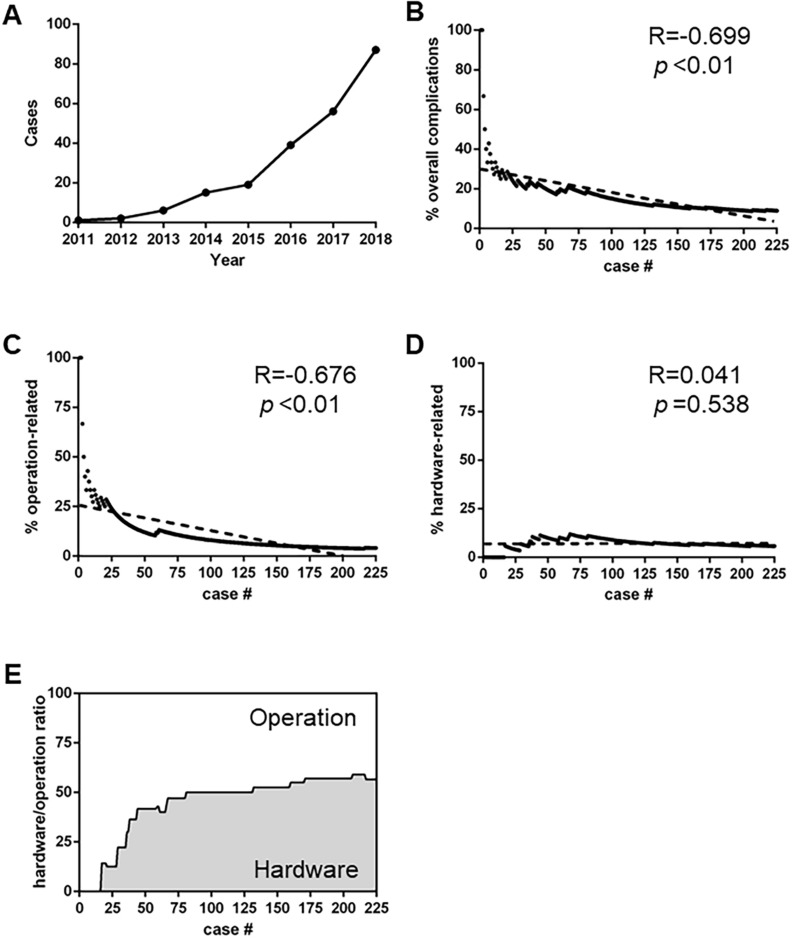
Secular trend of postoperative complications. **(A)**: The annual number of DBS surgical procedures in our center. **(B)** The incidence of overall complications with increased number of Deep brain stimulation (DBS) volumes (*R* = –0.699, *p* < 0.01) **(C,D)** Different trends of operation-related **(C)** and hardware-related **(D)** complications (*R* = –0.676, *p* < 0.01 and *R* = 0.041, *p* = 0.538, respectively). **(E)** The ratio of hardware-related complications versus operation-related complications.

In our cohort, an upward trend in the annual number of DBS surgeries was evident (Figure 3A). Meanwhile, the overall complication rate experienced a correspondingly dramatic fall (Figure 3B, *R* = −0.699, *P* < 0.01), which highlighted the importance of surgical experience in line with findings of some other studies (Falowski et al., 2015; Sorar et al., 2018). As shown in Figure 3C, the surgical experience was significantly related to the incidence of operation-related complications (*R* = −0.676, *P* < 0.01), rather than that of hardware-related complications (Figure 3D, *R* = 0.041, *P* = 0.538). Consequently, of all the complications, the proportion of hardware-related ones gradually increased (Figure 3E).

### Unplanned Readmission

Among 225 patients, 15 patients experienced unplanned readmissions (6.2%, Table 4). The average interval between primary surgery and readmission was 18.0 ± 19.2 months. Of these patients, 13 cases were admitted to the hospital due to complications; therefore, the complications were closely related to patient readmission (*R* = 0.730, *P* < 0.01). 12 out of 15 patients underwent surgery, including device reimplantation (*n* = 3), electrode revision (*n* = 2), extension wire replacement (*n* = 4), relief of stricture (*n* = 1), device removal (*n* = 1), and ventriculoperitoneal shunt (*n* = 1). Unsurprisingly, patients with complications were more likely to experience readmissions (*P* < 0.001).

**TABLE 4:** Causes for Unplanned Readmission and Interventions.

**Patient**	**Gender**	**Age**	**operation date**	**Readmission date**	**Interval (months)**	**Complication or not**	**Readmission reasons**	**Interventions**
1	F	52	2011/4/8	2017/4/21	72.3	Yes	Electrode misplace	Re-operation (L-STN→bi-GPi) on 2017/4/28
12	M	57	2014/4/17	2014/4/30	0.4	Yes	Symptomatic edema with transient loss of consciousness	Recovery after symptomatic treatment
17	M	56	2014/8/25	2016/11/1	26.2	Yes	Electrode misplace Extension wire high resistance	Removal of bilateral IPGs and extension wires on 2018/3/31
21	M	64	2014/12/4	2016/1/12	13.2	Yes	Respiratory distress	Re-operation on 2016/1/24
36	M	65	2015/10/29	2019/5/18	42.5	Yes	Extension wire fracture	Replacement of IPG and bilateral extension wires on 2019/5/20
38	M	53	2015/11/19	2017/11/29	24.3	Yes	Extension wire fracture	Replacement of bilateral extension wires on 2017/12/1
43	M	42	2015/12/1	2016/10/27	10.9	No	Programming issue	Reprogramming
44	M	64	2016/1/13	2018/3/22	26.2	Yes	Extension wire fracture after injury	Replacement of extension wire on 2018/4/3
59	F	68	2016/8/15	2016/9/2	0.6	Yes	Respiratory distress	Re-implantation of contralateral STN electrode and IPG on 2016/9/12
61	M	70	2016/8/20	2017/9/29	13.3	Yes	Hydrocephalus	ventriculoperitoneal shunt on 2017/10/13
66	F	55	2016/10/12	2017/5/17	7.1	Yes	Neck stricture formation	Relief of stricture on 2017/5/18
67	F	68	2016/10/20	2017/1/3	2.5	Yes	Electrode migration	Electrode adjustment on 2017/1/5
81	F	60	2016/12/29	2018/5/3	16.1	Yes	Electrode migration	Electrode adjustment on 2018/5/7
82	M	67	2016/12/31	2017/1/2	0.1	No	Severe pneumonia	Full recovery with antibiotics
160	M	43	2018/4/21	2019/6/10	13.6	Yes	Extension wire fracture after head injury	Replacement of right extension wire on 2019/6/12

*Abbreviations: GPi, globus pallidus internus; IPG, implantable pulse generator; STN, subthalamic nucleus.*

## Discussion

Deep brain stimulation surgery has drawn extensive attention regarding its safety issue (Rughani et al., 2013; Fenoy and Simpson, 2014; Fernandez-Pajarin et al., 2017; Rumalla et al., 2018). To date, several retrospective studies which investigated the incidence and risk factors for DBS-related complications have shown conflicting results, mainly due to differences in population characteristics, study design (PD alone or together with other movement disorders), cohort size, surgical experiences, and follow-up duration (Goodman et al., 2006; Baizabal Carvallo et al., 2012; Hu et al., 2017; Ramayya et al., 2017). Herein, we have reviewed a consecutive series of patients with advanced PD who underwent DBS surgery in a single center to eliminate most interference factors and reveal the incidence and risk factors of DBS-related complications in the Chinese population.

In this study, the statistical significance of early stage operation was demonstrated for operation-related complications. Practice makes perfect. Although it was reported that no DBS-related complication had be yet observed for the very first operations in a newly established neurosurgical center (Razmkon et al., 2019), our data confirmed the well-known merits of surgical experience on preventing postoperative complications, or more specifically, operation-related ones. We also observed a tendency that patients with one complication were more likely to present with another one at a later stage (Patient #7, 17, 59), which had been reported by Paluzzi et al. (2006). It is noteworthy that hardware-related complications were more common and problematic in our cohort, including wire fracture/high resistance (2.2%), electrode migration (0.89%), IPG migration (0.89%), and neck stricture formation (0.89%). There might be several reasons. First, the average follow-up duration was 45.2 months in this series, and a longer follow-up increased the cumulative probability of hardware-related complications (Oh et al., 2002). Second, although the ratio of hardware-related complications was higher, its incidence (13 out of 225 patients) still represented a lower rate than that in the most published studies. Third, the dualistic definitions of the operation- and hardware-related complications can be oversimplified, which causes ambiguity in categorizing certain types of adverse events. Nevertheless, the low incidence of the overall DBS-related complications shown in this study was quite encouraging.

Hypertension, as illustrated, is not only the most common comorbidity for patients with PD, but also a tremendous risk factor for operation-related complications in our cohort. Since it remains controversial whether hypertension increases the risk of postoperative complications (Yang et al., 2020), we have reviewed the complication details associated with hypertension. Interestingly, both patients (Patient #2, 12) who encountered severe peri-electrode edema were with hypertension (*P* = 0.0448, Fisher’s exact test). Severe peri-electrode edema is a rare, self-limited operation-related complication (Whiting et al., 2018), with the main symptoms of psychosis. Notwithstanding that the etiology of edema remains unknown (Deogaonkar et al., 2011; Nazzaro et al., 2017), we hence proposed that hypertension might mediate the symptomatic edema by the mechanisms of either CT-negative micro-hematoma or local cerebral perfusion pressure dysregulation. Also, we had a 79-year old patient with hypertension encountering acute heart failure and pneumonia soon after surgery (Patient #217). Therefore, the association between hypertension and postoperative complications could be more complex and extensive than previously assumed (Adogwa et al., 2017). Further studies in the larger cohorts should be conducted to determine whether hypertension is related to DBS-related complications.

Intracranial hemorrhage is a devastating operation-related complication. Retrospective studies reported the incidence of intracerebral hemorrhage between 0.6–6.0% (Binder et al., 2005; Park et al., 2011; Wang et al., 2017). In our series, one patient (0.44%) encountered intracranial hematoma who recovered without surgical intervention. Careful planning on preoperative contrast-enhanced MRI images and MER recording with single microelectrode are essential for avoiding vascular injury. Invasive arterial blood pressure should be controlled below 140/90mmhg during the entire course in our center.

Wound complications, especially infection, are among the most frequent and concerning hardware-related complications. While the incidence of infection ranged from 0 to 15.2% (Sixel-Doring et al., 2010; Bjerknes et al., 2014; Kim et al., 2017; Hardaway et al., 2018; Kantzanou et al., 2021), merely two patients (0.89%) experienced subcutaneous exudate in this cohort and were soon cured after local compression dressing and antibiotic administration. Regrading wound complications, our rationale is that the probability of such events can be minimized by the long-term efforts of a specialized operation team with strict enforcement of sterility, and reductant surgical modifications. Direct hardware implantation under the suture line should be meticulously avoided (Falowski et al., 2015). Alcohol-based solution was used for gross skin cleaning, and then povidone-iodine for formal incision preparation. Intraoperative irrigation with povidone-iodine and saline solutions before layer by layer closure with triclosan-coated absorbable sutures should be also noted.

There is sufficient discussion in the literature regarding the surgical technique issue (Fontaine et al., 2013; Linhares et al., 2013; Falowski et al., 2015; Rasouli and Kopell, 2016; White-Dzuro et al., 2016; Zhou et al., 2018); however, the relatively low incidence for postoperative complications in this cohort, especially infection, was attributed primarily to the reductant preventive approaches as we described, no matter how subtle or ordinary they were. For every complication requiring further surgery, there could be many more adverse events that were neglected. Therefore, it is reasonable and necessary to apply multiple surgical modifications. Meanwhile, device improvements, for instance, extension wire with high elasticity and corrosion resistance, and firmer electrode anchoring device, might also help lower the hardware-related risk.

## Limitations

There are some limitations to our study. First, this study was retrospective, non-randomized, and monocentric. For instance, most patients were Han Chinese in our study cohort, a population who were seldomly studied. Therefore, the results should be interpreted with caution. Second, the surgical modifications described above were gradually adopted during our practice, making it difficult to demonstrate the correlation between these modifications and the decrease of complication incidence. Third, with 23 adverse events in 20 cases, statistical power could be limited due to the low number of events per variable for logistic regression analysis (Peduzzi et al., 1996). Larger scale studies are necessary to solve this issue. Fourth, the association of DBS-related complications with quality of life (QoL) was not investigated in this study. Although the variable whether post-operation complications existed was not evaluated in a prognostic model to predict improvement in QoL following DBS surgery in patients with PD (Frizon et al., 2019), it would be helpful to understand the influence of DBS-related complications on QoL in patients with PD in the future.

## Conclusion

In summary, we demonstrated that hypertension was the most significant individual risk factor for DBS surgery, in contrast to surgical experience as a major iatrogenic factor. Stratified analysis validated these risk factors for operation-related complications. In contrast, no risk factor was identified for the hardware-related complications. These findings provide a new perspective to understanding the DBS-related complications for patients with PD, and highlight the importance of device improvement in lowering hardware-related adverse events.

## Data Availability Statement

The original contributions presented in the study are included in the article/supplementary material, further inquiries can be directed to the corresponding author/s.

## Ethics Statement

The studies involving human participants were reviewed and approved by the Medical Ethical Committee, Qilu Hospital of Shandong University. Written informed consent for participation was not required for this study in accordance with the national legislation and the institutional requirements.

## Author Contributions

SX performed the acquisition, analysis and interpretation of data, and wrote the manuscript. WW performed the statistical analysis. SC and QW participated in the acquisition of data. CL and XM provided with valuable feedback on the manuscript. WL and TC provided with valuable feedback about the technique modifications. SjX designed and supervised the study, and conducted the final approval of the manuscript. All authors reviewed the manuscript.

## Conflict of Interest

The authors declare that the research was conducted in the absence of any commercial or financial relationships that could be construed as a potential conflict of interest.
